# Age and Procedural Timing for Asymptomatic Severe Aortic Stenosis: Analysis From the EARLY TAVR Trial

**DOI:** 10.1161/CIRCINTERVENTIONS.125.016370

**Published:** 2026-06-03

**Authors:** Kashish Goel, Brian R. Lindman, Allan Schwartz, David J. Cohen, Gennaro Giustino, J. Bradley Oldemeyer, Justin Strote, Vasilis Babaliaros, Chandan M. Devireddy, Michael P. Fischbein, William F. Fearon, David Daniels, Christian Spies, Adnan K. Chhatriwalla, Hussam S. Suradi, Pinak Shah, Molly Szerlip, Thom Dahle, Dimitrios Apostolou, Raj Makkar, Charles J. Davidson, Tej Sheth, Paul Sorajja, James T. DeVries, Jeffrey Southard, Jeremiah P. Depta, Andrei Pop, Michael J. Rinaldi, Salem Badr, Mathew R. Williams, Mark J. Russo, Mayra Guerrero, James M. McCabe, Philippe Pibarot, Yizhuo Wang, Martin B. Leon, Philippe Généreux

**Affiliations:** 1 Division of Cardiovascular Medicine (K.G., B.R.L.), Vanderbilt University Medical Center, Nashville, TN.; 2 Structural Heart and Valve Center (B.R.L.), Vanderbilt University Medical Center, Nashville, TN.; 3 Columbia University Medical Center/NewYork-Presbyterian Hospital (A.S., D.J.C., M.B.L.).; 4 Cardiovascular Research Foundation, New York, NY (D.J.C., M.B.L.).; 5 St. Francis Hospital and Heart Center, Roslyn, NY (D.J.C.).; 6 Gagnon Cardiovascular Institute, Morristown Medical Center, NJ (G.G., P.G.).; 7 University of Colorado Health, Loveland (J.B.O., J. Strote).; 8 Structural Heart and Valve Center, Emory University School of Medicine, Atlanta, GA (V.B., C.M.D.).; 9 Department of Cardiothoracic Surgery, Stanford University School of Medicine, CA (M.P.F., W.F.F.).; 10 Division of Cardiology, California Pacific Medical Center, San Francisco (D.D., C.S.).; 11 Saint Luke’s Mid America Heart Institute, Kansas City, MO (A.K.C.).; 12 Division of Cardiology, Rush University Medical Center, Chicago, IL (H.S.S.).; 13 Division of Cardiovascular Medicine, Brigham and Women’s Hospital, Boston, MA (P. Shah).; 14 Baylor Scott and White The Heart Hospital Plano, TX (M.S.).; 15 CentraCare Heart & Vascular Center, St. Cloud, MN (T.D.).; 16 Center for Structural Heart Disease, Henry Ford Health System, Detroit, MI (D.A.).; 17 Smidt Heart Institute, Cedars-Sinai Medical Center, Los Angeles, CA (R.M.).; 18 Northwestern University Feinberg School of Medicine, Chicago, IL (C.J.D.).; 19 Population Health Research Institute, McMaster University and Hamilton Health Sciences, Ontario, Canada (T.S.).; 20 Minneapolis Heart Institute Foundation, Abbott Northwestern Hospital, MN (P. Sorajja).; 21 Heart and Vascular Center, Section of Cardiovascular Medicine, Dartmouth-Hitchcock Medical Center, Lebanon, NH (J.T.D.V.).; 22 Division of Cardiovascular Medicine, UC Davis Health System, University of California-Davis, Sacramento (J. Southard).; 23 Sands-Constellation Heart Institute, Rochester Regional Health, NY (J.P.D.).; 24 Department of Cardiology, AMITA Alexian Brothers Medical Center, Elk Grove Village, IL (A.P.).; 25 Atrium Health Sanger Heart & Vascular Institute, Charlotte, NC (M.J. Rinaldi).; 26 Prairie Education and Research Cooperative, Springfield, IL (S.B.).; 27 New York University Langone Medical Center (M.R.W.).; 28 Rutgers-Robert Wood Johnson Medical School, New Brunswick, NJ (M.J. Russo).; 29 Department of Cardiovascular Medicine, Mayo Clinic, Rochester, MN (M.G.).; 30 Department of Cardiology, Beth Israel Deaconess Medical Center, Boston, MA (J.M.M.C.).; 31 Department of Cardiology, Québec Heart and Lung Institute, Laval University, Canada (P.P.).; 32 Edwards Lifesciences, Irvine, CA (Y.W.).

**Keywords:** age groups, aortic valve stenosis, asymptomatic diseases, stroke, transcatheter aortic valve replacement

## Abstract

**BACKGROUND:**

The EARLY TAVR trial (Evaluation of TAVR Compared to Surveillance for Patients with Asymptomatic Severe Aortic Stenosis) demonstrated that early transcatheter aortic valve replacement (TAVR) was superior to clinical surveillance (CS) in asymptomatic severe aortic stenosis. The relative impact of early TAVR versus CS strategy by age is unknown.

**METHODS:**

The study population of the EARLY TAVR trial was stratified into 4 age groups: 65 to 69 years (n=141), 70 to 74 years (n=263), 75 to 79 years (n=250), and ≥80 years (n=247). Associations between age and the trial primary end point of death, stroke, or unplanned cardiovascular hospitalization; the composite end point of death, stroke, or heart failure hospitalization; and its individual components were examined. Interaction tests evaluated whether the treatment effect of early TAVR versus CS differed by age.

**RESULTS:**

No interaction was detected between age and the treatment effect of early TAVR versus CS for the composites or individual outcomes. We observed lower stroke rates with early TAVR compared with CS in the youngest (65–69 years, absolute risk reduction, 13%; *P*=0.008) and oldest (≥80 years; absolute risk reduction, 12.3%; *P*=0.029) age groups. The absolute difference in heart failure hospitalization rates between the early TAVR and CS arms at 2 years was greatest in the oldest patients (≥80 years, 9.1%; 75–79 years, 5.9%; 70–74 years, 5.1%; 65–69 years, 4.4%). In the CS group, time to conversion to AVR was similar across all age groups (median, 11 months; *P*=0.73). Approximately one-third of younger patients (65–69 years) in the CS arm presented with acute valve syndrome at the time of conversion, the frequency of which tended to increase with age (*P*=0.06).

**CONCLUSIONS:**

In the EARLY TAVR trial, the relative benefit of early TAVR over CS was consistent among all age groups. The greatest absolute reduction in stroke rate with early TAVR compared with CS appeared in the youngest and oldest groups, whereas reduction in heart failure hospitalization was most pronounced in the oldest patients. These data suggest that early TAVR should be considered in all age groups above 65 years.

**REGISTRATION:**

URL: https://www.clinicaltrials.gov; Unique identifier: NCT03042104.

**What is Known**

- The EARLY TAVR trial (Evaluation of TAVR Compared to Surveillance for Patients with Asymptomatic Severe Aortic Stenosis) reported that early intervention in asymptomatic severe aortic stenosis led to improved outcomes across multiple outcomes.
- Prior surgical trials in asymptomatic severe aortic stenosis enrolled younger patients, and age influences clinical decisions regarding the timing of intervention given its impact on lifetime management.

**What the Study Adds**

- This age-stratified analysis of the EARLY TAVR trial showed no interaction between age and treatment effect, suggesting that the benefit of early intervention in asymptomatic severe aortic stenosis was consistent across all age groups ≥65 years.
- The stroke benefit of early transcatheter aortic valve replacement was most prominent in the younger (65–69 years) and the older (≥80 years) groups, whereas the reduction in heart failure hospitalization was most pronounced in the older group.
- Given the consistent benefit and lack of harm observed across all age groups, a strategy of early transcatheter aortic valve replacement should be considered as an alternative to clinical surveillance among patients aged ≥65 years with asymptomatic severe aortic stenosis.

Age is a key component of the overall risk stratification in patients with aortic stenosis (AS) and influences the choice between transcatheter aortic valve replacement (TAVR) and surgical aortic valve replacement (AVR) in symptomatic patients. Age may also affect decisions regarding timing of intervention as it relates to lifetime risk and patient management. EARLY TAVR (Evaluation of TAVR Compared to Surveillance for Patients with Asymptomatic Severe Aortic Stenosis) and 3 other trials evaluating early intervention in asymptomatic severe AS included a broad range of patient ages.^1–4^ Taken together, these trials indicate potential improvement in outcomes with early intervention in asymptomatic severe AS, regardless of age.^5^

Nonetheless, questions remain regarding the generalizability of the findings, particularly among the younger and older ends of the age spectrum. In younger patients, clinicians may prefer to delay intervention until symptom onset, assuming that the early treatment would initiate an earlier cycle of potential repeat valve interventions later in life; these considerations may lead to a preference for clinical surveillance (CS) in younger patients despite the overall trial results. For older patients, one may speculate that the risks of early intervention may be greater, or that the benefit of early intervention may be reduced due to shorter life expectancy; these considerations may bias the clinician to pursue CS in the older patients.

Accordingly, we performed a post hoc, age-stratified analysis of the largest randomized trial to date in this patient population to provide insights into these clinically important questions. We hypothesized that the benefit of an early TAVR strategy would be consistent across age groups in the EARLY TAVR trial.

## Methods

### Data Availability

The data for this study will not be made available to other researchers for the purpose of replicating the results.

### Trial Design and Patient Population

EARLY TAVR is a prospective, multicenter, open-label trial that randomized asymptomatic patients with severe AS 1:1 to either transfemoral TAVR, using a SAPIEN 3 or SAPIEN 3 Ultra valve (Edwards Lifesciences), or CS.^1^ All randomized patients were included in the intent-to-treat analysis. The trial design, rationale, and eligibility criteria have been described previously.^1,6^ The institutional review boards of each site approved the trial protocol and all patients provided written informed consent.

### Clinical Outcomes

The primary end point of the EARLY TAVR trial was a nonhierarchical composite of all-cause death, stroke, or unplanned cardiovascular hospitalization, as defined previously.^1^ Prespecified secondary end points are detailed in the Supplemental Methods. Additional exploratory outcomes included a composite of death, stroke, unplanned cardiovascular hospitalization (not including early AVR conversions), or AVR with acute valve syndrome as well as a composite of death, stroke, or heart failure hospitalization (HFH), as defined previously.^1^ In the CS arm, timing of conversion to AVR and type of clinical presentation at the time of conversion (ie, advanced or acute signs and symptoms [acute valve syndrome], progressive signs and symptoms [progressive valve syndrome], or no symptoms [silent syndrome], as defined previously)^1^ were evaluated in each age group. All end points were adjudicated by the clinical events committee based on the prespecified definitions and without blinding to treatment assignment.

### Statistical Analysis

Patients were stratified into 4 age groups for this analysis: 65 to 69, 70 to 74, 75 to 79, and ≥80 years (Figure S1). Age quartiles were also assessed as a sensitivity analysis. Continuous variables for TAVR versus CS arms were compared using the Wilcoxon rank-sum test and Jonckheere-Terpstra test for comparing 4 ordered age groups; binary outcomes for TAVR versus CS arms were compared using Fisher exact test and the Cochran-Armitage trend test for comparing 4 ordered age groups. Time-to-event outcomes are presented as Kaplan-Meier curves and were compared using log-rank tests. The treatment effect size was evaluated using hazard ratios and 95% CIs. Homogeneity of treatment effect by age group was assessed using an unadjusted Cox proportional hazards model with the interaction term. A *P*<0.05 (*P*<0.1 for interaction tests) was considered statistically significant (without correction for multiple comparisons). For all outcomes of interest, hazard ratios along with incidence rates per 100 person-years up to 5 years and numbers needed to treat at 2 years are presented in forest plots. All statistical analyses were performed using SAS software, version 9.4 (SAS Institute, Cary, NC) and R, version 4.2.0.

## Results

Of 901 patients enrolled in EARLY TAVR, 141 (15.6%) were aged 65 to 69 years, 263 (29.2%) were 70 to 74 years, 250 (27.7%) were 75 to 79 years, and 247 (27.4%) were ≥80 years. Baseline characteristics by age group are presented in Table 1. Older patients had a higher frequency of comorbidities, whereas younger patients had a higher percentage of bicuspid morphology. Echocardiographic parameters, including peak aortic jet velocity, mean gradient, left ventricular (LV) ejection fraction, and LV mass index, were similar across all age groups. In the overall population, older age was associated with a higher rate of death (*P*=0.024), HFH (*P*=0.012), and the combined rate of death, stroke, or HFH (*P*=0.005). There was no difference in the stroke rate between the age groups (Figure S2).

**Table 1.**
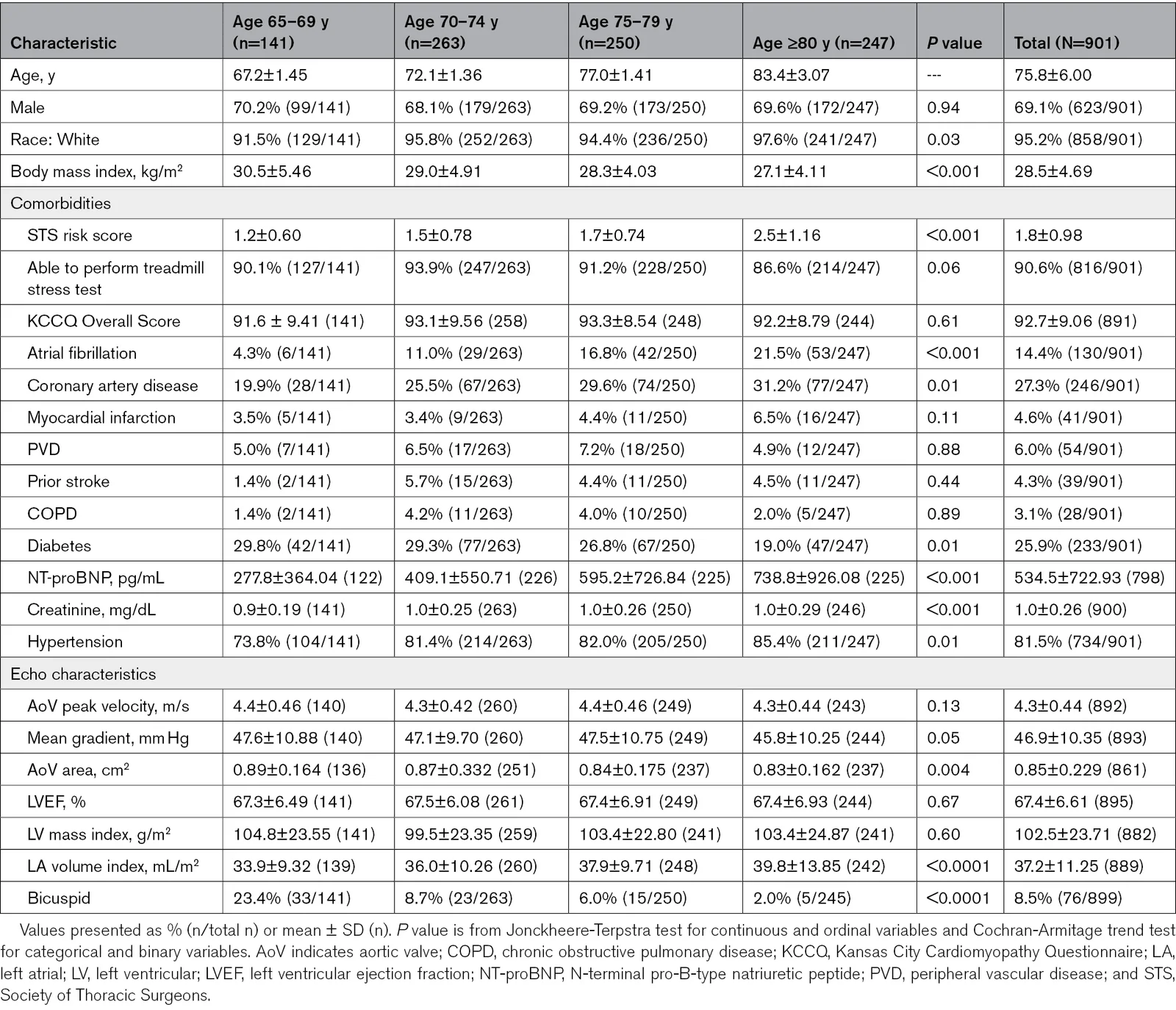
Baseline Characteristics Stratified by Age

### Early TAVR Versus CS by Age Group

Baseline and echocardiographic characteristics were similar between the TAVR and CS arms across age groups (Table S1). Procedural characteristics and periprocedural outcomes are presented in Tables S2 and S3.

Over a median follow-up of 3.8 years, the rates of the primary end point of death, stroke, or unplanned cardiovascular hospitalization were significantly lower in the early TAVR arm compared with CS, with no variation in treatment effect by age group (interaction *P*=0.47; Figure 1). Similarly, there was no significant interaction by age for the composite of death, stroke, unplanned cardiovascular hospitalization (not including early AVR conversions) or intervention/reintervention with acute valve syndrome (interaction *P*=0.45), and with respect to the composite of death, stroke, or HFH (interaction *P*=0.53). There was no heterogeneity of treatment effect for the 3 composite end points when the study population was stratified by age quartiles (Figure S3). Composite outcomes by age group in the early TAVR and CS arms are presented in Figures S4 and S5.

**Figure 1.**
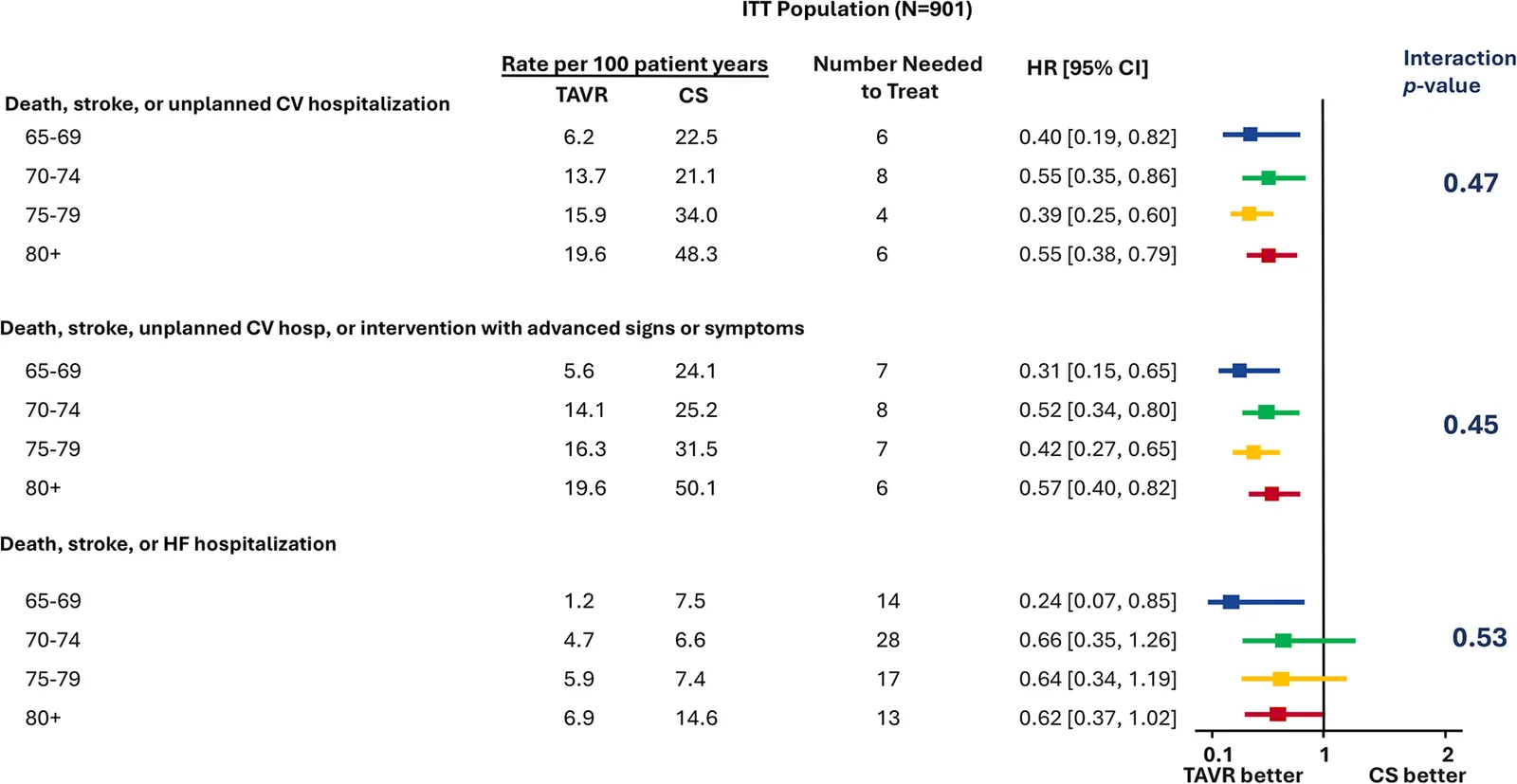
**Interaction terms by treatment and age for composite outcomes.** Forest plot showing clinical event rates per 100 patient-years, number needed to treat (NNT; calculated using the 2-year Kaplan-Meier rate), risk, and interaction *P* values for 3 composite end points according to the overall intent-to-treat (ITT) population and 4 age groups. Clinical event hazard ratios (HRs) and 95% CIs are based on Cox proportional hazards models with data up to 5 years. Interaction *P* values reflect the interaction between age group and treatment arm. CS indicates clinical surveillance; CV, cardiovascular; HF, heart failure; and TAVR, transcatheter aortic valve replacement.

No significant interaction by age group was observed for the individual outcomes of death (interaction *P*=0.81), stroke (interaction *P*=0.17), and HFH (interaction *P*=0.52; Figure 2). Rates of all-cause death were similar between the early TAVR and CS arms across all age groups (Figure 3). Although the interaction term for stroke was not significant, a substantially lower rate was observed with early TAVR compared with CS in the youngest and oldest subgroups (Figure 4). Specifically, among patients aged 65 to 69 years, no strokes occurred in the early TAVR arm during the follow-up period, whereas in the CS arm, 7 strokes occurred among 69 patients (*P*=0.008; Figure 4A). Among those aged ≥80 years, the stroke rate was also lower with early TAVR compared with CS (*P*=0.029; Figure 4D). For HFH rates, the absolute difference between early TAVR and CS was numerically most pronounced in the oldest age group (≥80 years) at 9.1% at 2 years, with a number needed to treat of 11 patients to prevent 1 HFH event (Figures 2 and 5).

**Figure 2.**
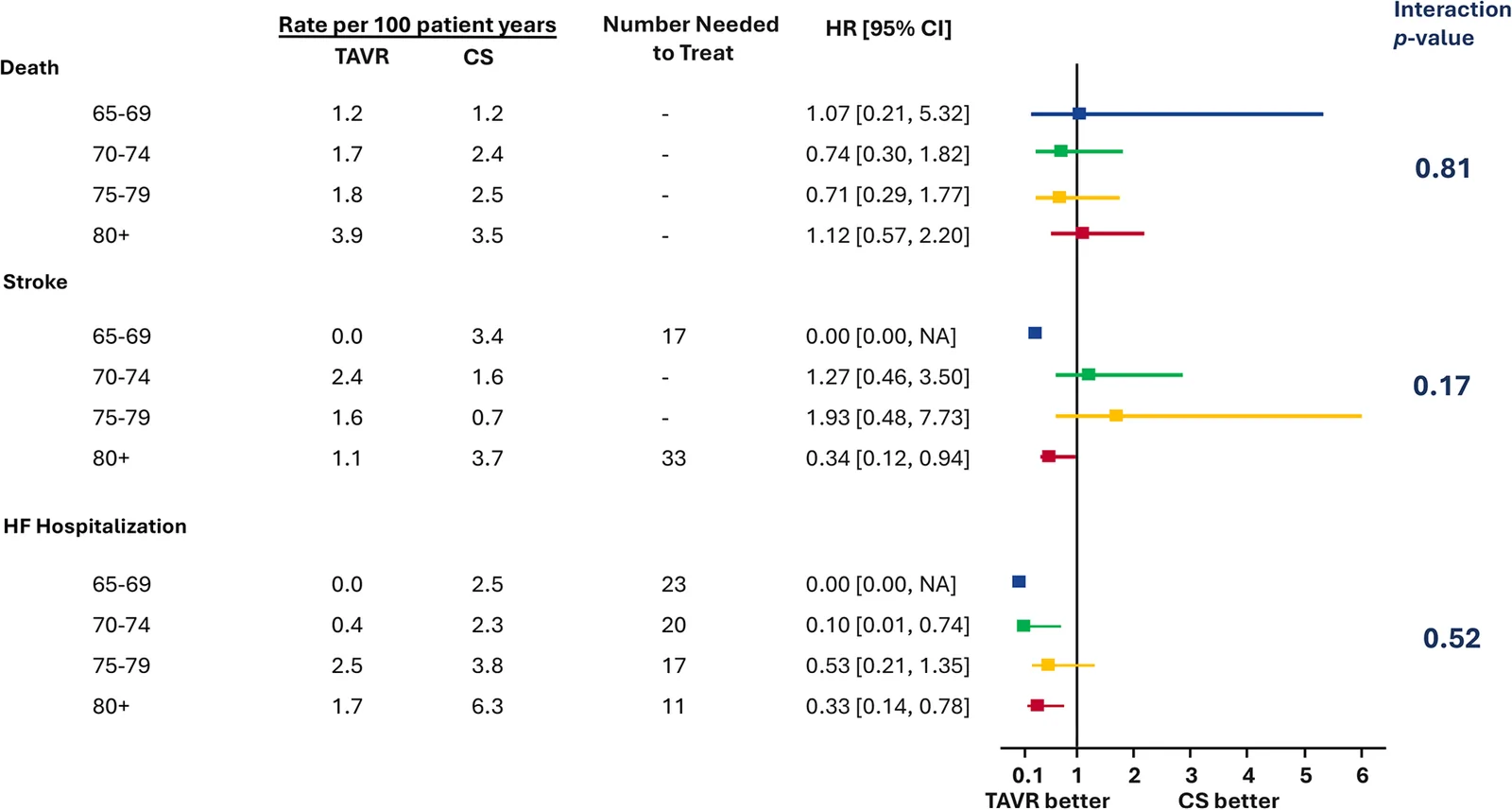
**Interaction terms by treatment and age for individual outcomes.** Forest plot showing clinical event rates per 100 patient-years, number needed to treat (NNT; calculated using the 2-year Kaplan-Meier rate), risk, and interaction *P* values for the individual outcomes of death, stroke, and heart failure (HF) hospitalization according to the overall intent-to-treat (ITT) population and 4 age groups. Clinical event hazard ratios (HRs) and 95% CIs are based on Cox proportional hazards models with data up to 5 years. Interaction *P* values reflect the interaction between age group and treatment arm. CS indicates clinical surveillance; NA, not available; and TAVR, transcatheter aortic valve replacement.

**Figure 3.**
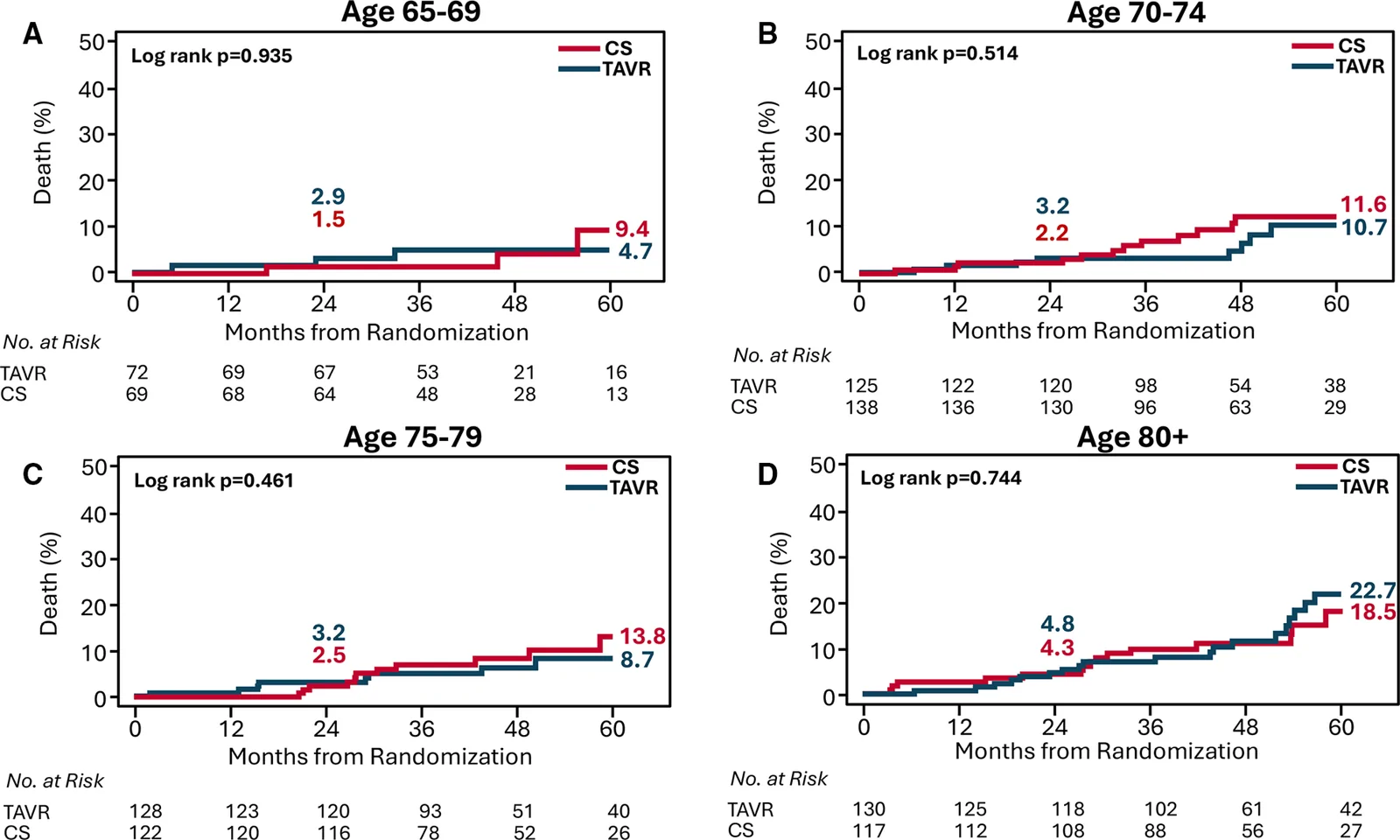
**Kaplan-Meier (KM) curves for death stratified by age and treatment.** KM curves for death in the 65 to 69 years (**A**), 70 to 74 years (**B**), 75 to 79 years (**C**), and ≥80 years (**D**) age groups. *P* values shown are based on unadjusted log-rank tests with data up to 5 years. CS indicates clinical surveillance; and TAVR, transcatheter aortic valve replacement.

**Figure 4.**
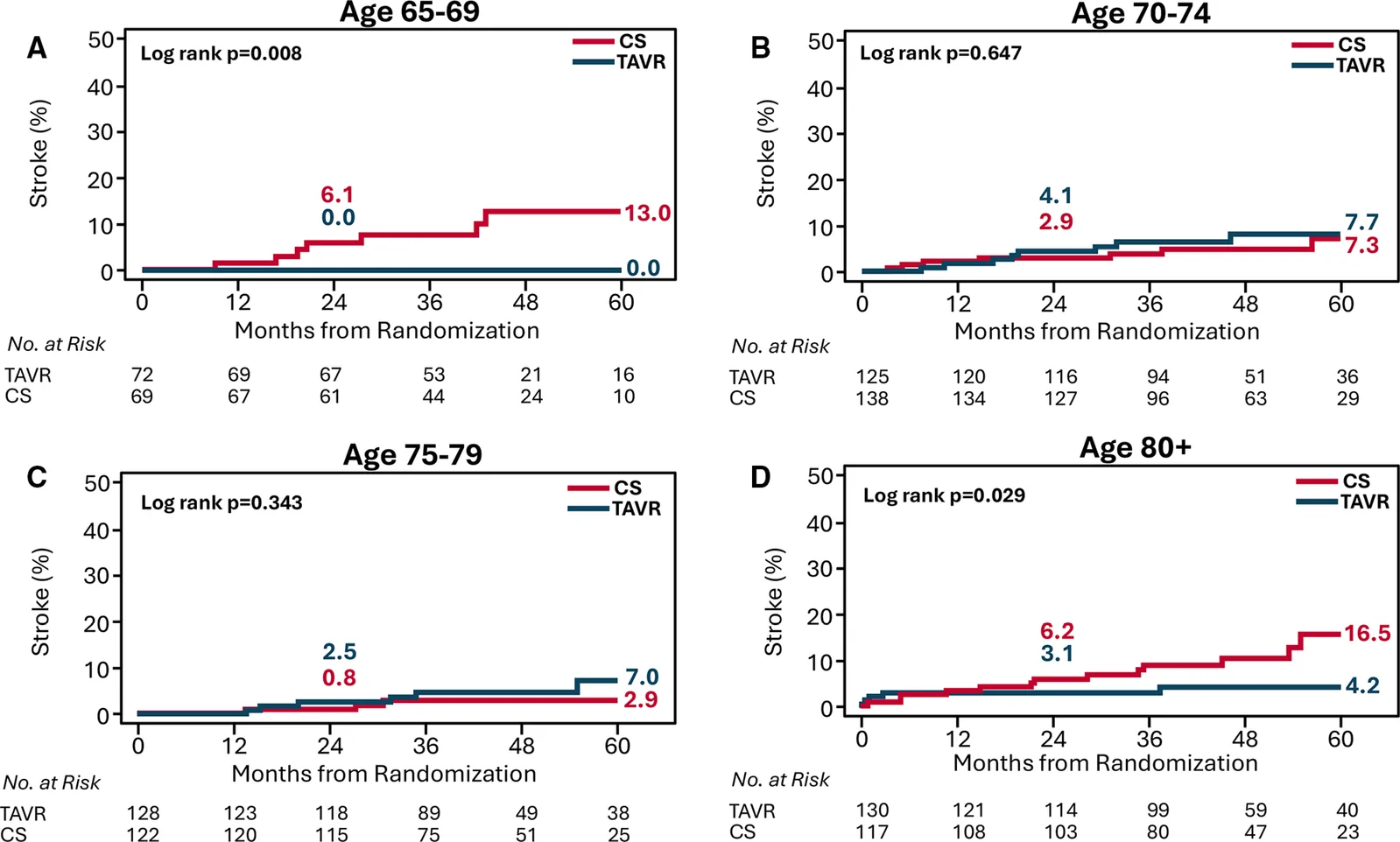
**Kaplan-Meier (KM) curves for stroke stratified by age and treatment.** KM curves for stroke in the 65 to 69 years (**A**), 70 to 74 years (**B**), 75 to 79 years (**C**), and ≥80 years (**D**) age groups. *P* values shown are based on unadjusted log-rank tests with data up to 5 years. CS indicates clinical surveillance; and TAVR, transcatheter aortic valve replacement.

**Figure 5.**
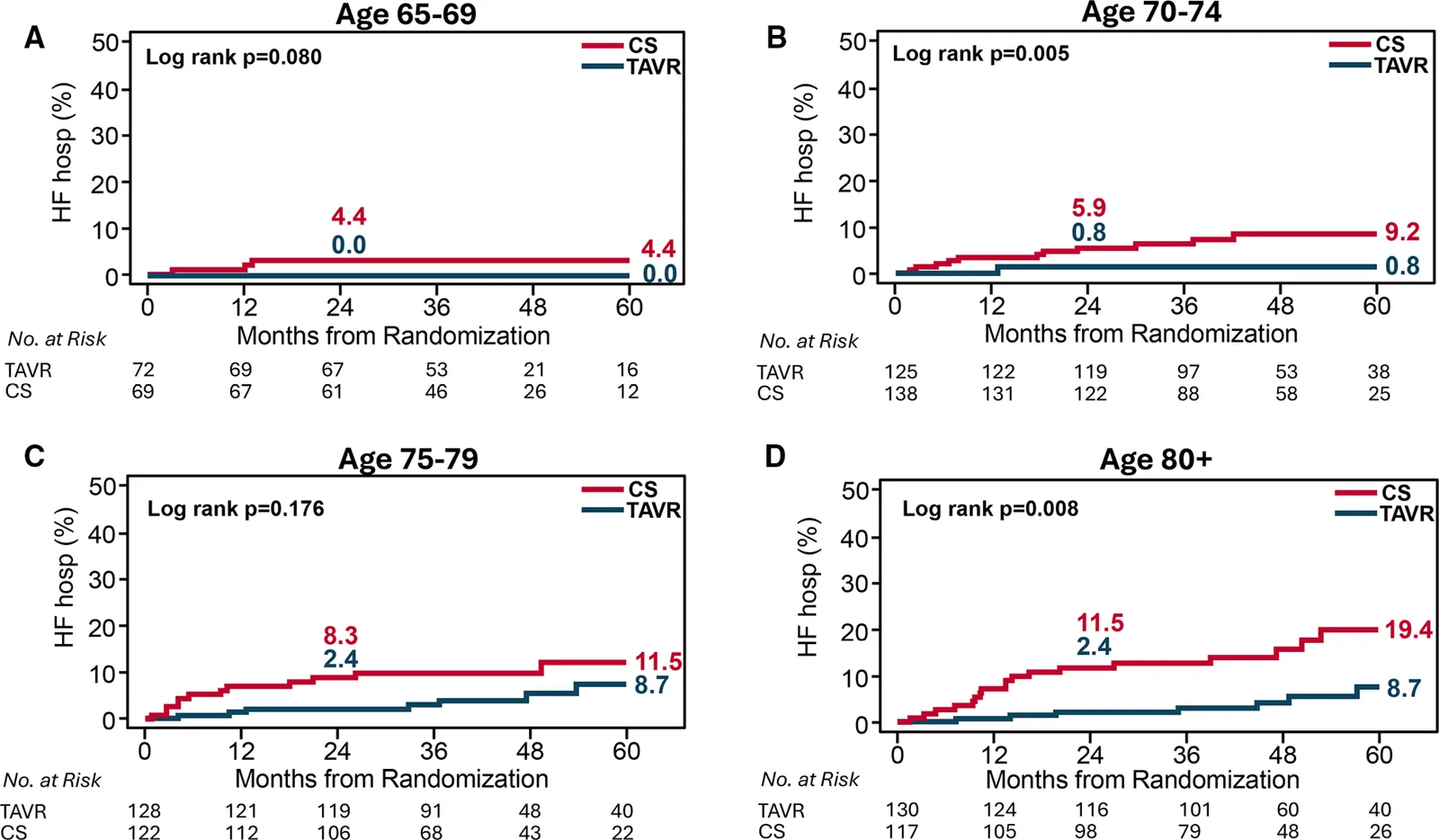
**Kaplan-Meier (KM) curves for heart failure (HF) hospitalization stratified by age and treatment.** KM curves for HF hospitalization in the 65 to 69 years (**A**), 70 to 74 years (**B**), 75 to 79 years (**C**), and ≥80 years (**D**) age groups. *P* values are based on unadjusted log-rank tests with data up to 5 years. CS indicates clinical surveillance; and TAVR, transcatheter aortic valve replacement.

### AVR Conversion in the CS Arm

In the CS arm, the time to conversion to AVR did not differ across age strata (*P*=0.73; Figure 6A). The median time to conversion was similar among the younger and older patients (65–69 years, 13.0 months; 70–74 years, 11.0 months; 75–79 years, 9.6 months; and ≥80 years, 11.7 months). Older patients tended to have a higher likelihood of conversion with acute valve syndrome (*P*=0.06; Figure 6B); however, even among the youngest group, 32.3% of patients presented with acute valve syndrome at the time of AVR.

**Figure 6.**
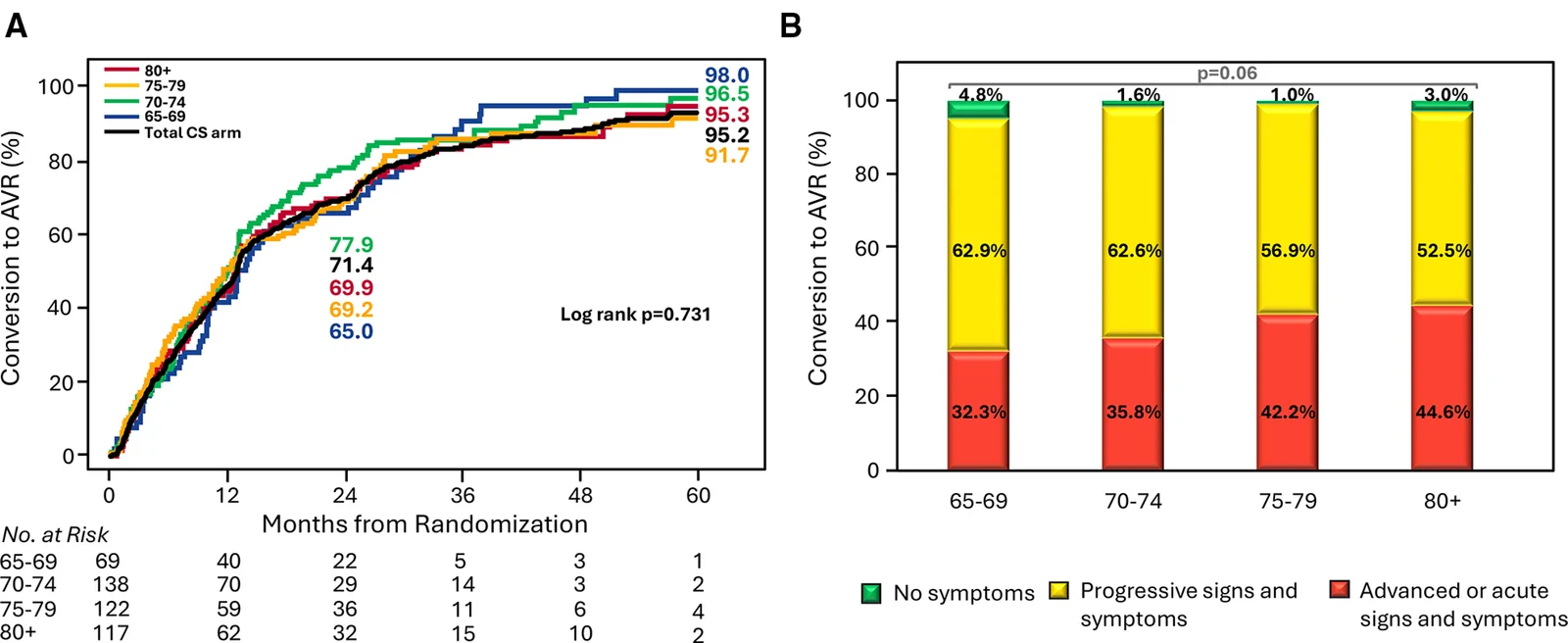
**Timing and clinical presentation class of aortic valve replacement (AVR) in the clinical surveillance (CS) arm across different age groups. A**, Kaplan-Meier curves for the timing of conversion to AVR in patients randomized to the CS arm across 4 age groups and in the overall CS arm. **B**, Stacked bar graphs showing the clinical presentation class at the time of AVR conversion according to 4 age groups in the CS arm. *P* values are based on Jonckheere-Terpstra trend tests.

### Functional and Echocardiographic Outcomes

The proportion of patients who were alive and well over 2 years was significantly greater in the early TAVR arm compared with CS across all age groups (Table 2). The integrated measure of LV health at 2 years was significantly higher in early TAVR versus CS in the 65 to 69 age group (63.9% versus 28.3%; *P*=0.0002) and was numerically higher with early TAVR across the remaining age groups, although these differences were not statistically significant (Table 2).

**Table 2.**
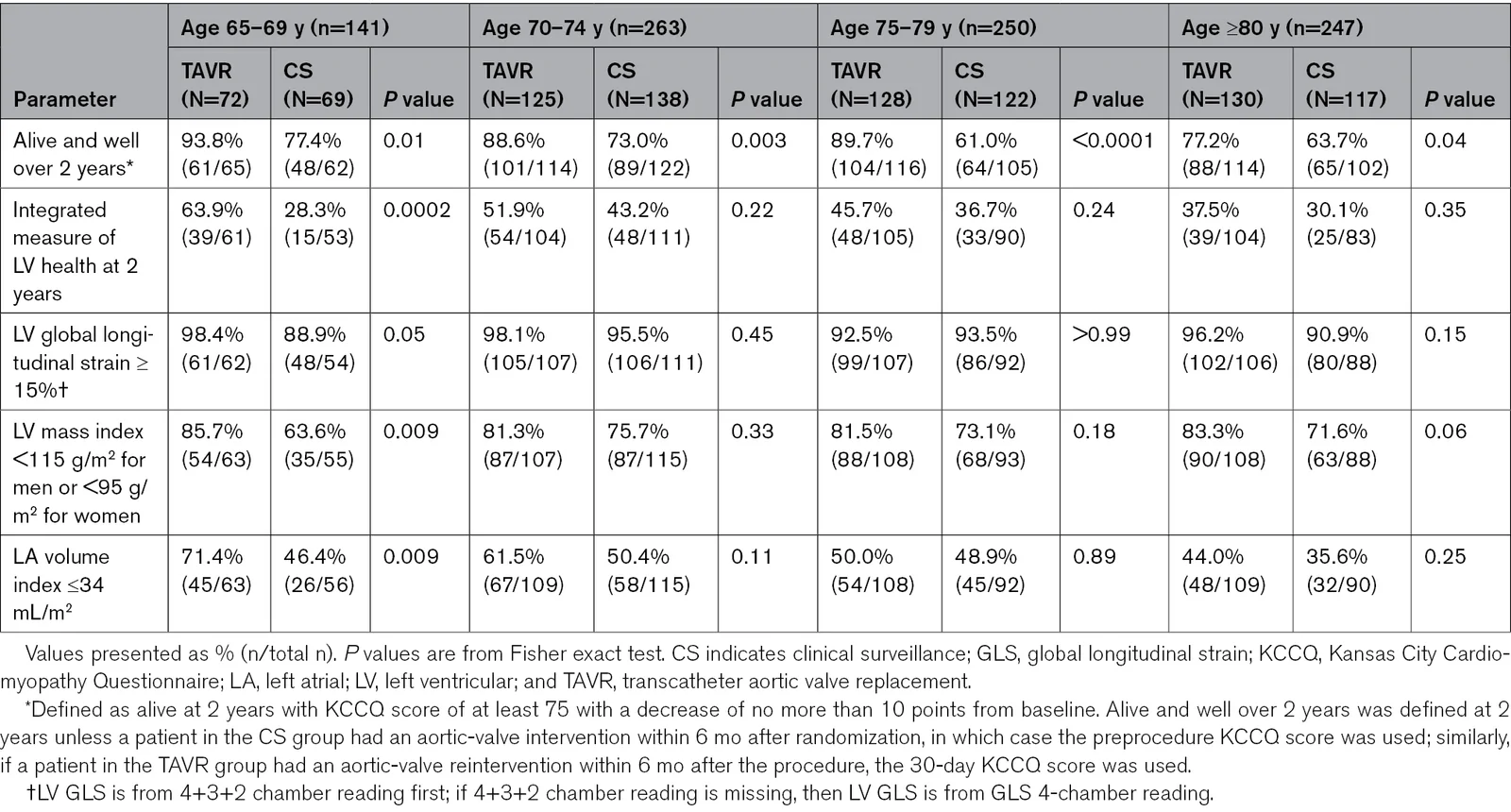
Alive and Well Over 2 Years and LV Health Stratified by Age and Treatment

## Discussion

This post hoc, age-stratified analysis of the EARLY TAVR trial revealed several important findings. The relative benefit of early intervention with TAVR compared with CS was consistent across age groups for the primary end point and several exploratory composite outcomes. While relative treatment effects were consistent across age strata, differences in absolute event rates were observed: HFH reduction was most prominent among the oldest patients (≥80 years), and stroke reduction was most notable in the youngest (65–69 years) and oldest (≥80 years) patients. Median time to AVR conversion was similar across age groups, and at least one-third of patients presented with acute valve syndrome, most often in older patients.

The primary results of the EARLY TAVR trial reported a significant benefit of early intervention in asymptomatic severe AS.^1^ One may hypothesize that this benefit may have been derived primarily from the older patients in the study, given the mean age of 75.8 years. Younger patients could potentially remain asymptomatic for longer and undergo intervention later in life. However, 2 prior randomized trials in younger patients with a mean age of 64 to 67 years demonstrated a significant reduction in mortality with early surgical AVR compared with CS.^2,3^ The large sample size of EARLY TAVR enabled the inclusion of 141 patients in the younger age group, similar to the number of patients in the RECOVERY (Randomized Comparison of Early Surgery versus Conventional Treatment in Very Severe Aortic Stenosis; n=145) and AVATAR (Aortic Valve Replacement Versus Conservative Treatment in Asymptomatic Severe Aortic Stenosis; n=157) trials. Our results suggest that the clinical benefit of early TAVR compared with CS was consistent among all age groups, including younger patients (65–69 years), as evidenced by a lower risk of the composite of death, stroke, or HFH in this age group (hazard ratio, 0.24 [95% CI, 0.07–0.85]). These findings complement and reinforce the results of RECOVERY and AVATAR. With respect to stroke, early TAVR had a 0% stroke occurrence compared with 10% in the CS group in patients aged 65 to 69 years during follow-up. Contrary to our expectations, the time to AVR conversion in the CS arm was similar across age groups. In addition, one-third of the patients in the younger age group presented with acute valve syndrome, which is associated with worse outcomes.^7–9^ This has important implications for lifetime management in younger patients, who are likely to live longer, and the benefits of early intervention may be even greater over extended follow-up. Overall, the combination of prior studies and the current analysis reinforces that early intervention yields better outcomes in younger patients with asymptomatic severe AS.

Older age was associated with increased risks of mortality and HFH, reflecting a higher comorbidity burden. Concerns regarding procedural complications and shorter life expectancy may influence the timing of early intervention in older patients. However, among patients aged ≥80 years, no differences in periprocedural complications were observed between early TAVR and CS patients undergoing deferred AVR at the time of symptom onset. In this age group, early intervention was associated with significant reductions in stroke and HFH compared with CS. Because the relative benefit of early intervention did not differ across age groups and overall event rates were higher among older patients, the absolute benefit of early intervention was numerically more pronounced in this oldest group. The 9.1% absolute reduction in HFH rates at 2 years with early intervention compared with CS in patients aged ≥80 years, corresponding to a number needed to treat of 11 to prevent 1 HFH event, is consistent with prior randomized clinical trials evaluating early intervention in asymptomatic severe AS.^2–4^ This may reflect untreated AS resulting in worsening myocardial fibrosis, LV hypertrophy, hemodynamics, and LV function, potentially resulting in irreversible cardiac damage.^10–13^ Although early intervention was not associated with a lower hazard of death, the reductions in stroke and HFH—while hypothesis-generating—may be clinically meaningful to patients, families, and the health care system, as these clinical consequences lead to significant morbidity and health care costs.

Patients are most fearful of stroke in terms of complications because of the potential disability associated with it, and this can be even more alarming at a younger age or lead to significant disability in older patients. The stroke reduction with early TAVR in the younger and older patients is hypothesis-generating and may reflect an increased risk of ischemic stroke associated with untreated AS in age- and sex-matched populations.^14^ Possible biologic explanations include a reduced risk of calcific emboli from the valve after TAVR due to reduction of high shear stress and a reduced rate of AS-related atrial fibrillation and subsequent strokes.^14^ Atrial fibrillation could potentially impact the stroke rates; however, the sample size in each age group was too small for detailed analysis. Patients aged 65 to 69 years had a significantly higher proportion of bicuspid valves, which are usually more calcified. It could be hypothesized that treating this group earlier may reduce embolic risk. This differs from surgery, where the postoperative risk of atrial fibrillation is much higher. These findings call for further attention to the relationship between AS and stroke and the role of early intervention in reducing stroke risk in patients with AS. The reason for the biphasic (youngest and oldest age groups only) stroke reduction with early intervention is unclear and needs further investigation.

### Limitations

This post hoc analysis has inherent limitations. Findings should be considered hypothesis-generating, as no corrections were made for multiple comparisons. Small age-group sample sizes limit power, although they are comparable to those in prior trials.^2,3^ Inclusion was limited to patients ≥65 years, reducing generalizability to patients under 65 years. The study includes balloon-expandable valves; therefore, the results may not be applicable to other valve types. Low mortality rates rendered age-stratified mortality analyses underpowered, and longer follow-up is needed to assess any long-term survival benefits.

### Conclusions

The age-stratified analysis of the EARLY TAVR trial suggests that the clinical benefit of early TAVR compared with CS in asymptomatic severe AS is consistent among all patient age groups ≥65 years of age. Although there was no evidence of effect modification by age, hypothesis-generating observations indicate the relative and absolute benefit of early intervention for stroke in the youngest and oldest patients. The median time to conversion was similar across all age groups, and approximately one-third of patients in the CS arm presented with acute valve syndrome at the time of intervention. Collectively, these findings reinforce the overall results of the trial by demonstrating that the benefit of early intervention extends across the age spectrum. Future studies should evaluate the long-term impact of early valve intervention on stroke in asymptomatic severe AS.

## Article Information

### Disclosures

Dr Goel reports consulting fees from Abbott Vascular, Edwards Lifesciences, Teleflex, and Nyra Medical and consulting fees and stock options from Trisol Laplace. Dr Lindman reports consulting fees from Anteris, AstraZeneca, Medtronic, and Kardigan; consulting fees and institutional grant/contracts from Edwards Lifesciences; and support from R01HL164526 and R01AG073633 from the NIH. Dr Cohen reports consulting fees and institutional grant/contracts from Abbott Vascular, Boston Scientific Corporation, Edwards Lifesciences, and Medtronic and institutional grant/contracts from JenaValve. Dr Giustino reports consulting fees from Medtronic and Edwards Lifesciences and equity in Antegrade Medical, Inc. Dr Oldemeyer serves as an investigator, Case Review Committee member, and Steering Committee member for Edwards Lifesciences. Dr Babaliaros reports consulting fees from Abbott Vascular and Edwards Lifesciences and stock options from TransMural Systems. Dr Devireddy reports consulting fees from Medtronic and Recor Medical. Dr Fearon reports institutional grant/contracts from Abbott Vascular, CathWorks, Inc, and Medtronic; consulting fees from ShockWave and Edwards Lifesciences; stock options from HeartFlow, Inc; and employment with Veterans Affairs Palo Alto. Dr Daniels and Dr Spies report consulting fees from Edwards Lifesciences. Dr Chhatriwalla reports consulting and speaker fees from Abbott Vascular, Edwards Lifesciences, and Medtronic and research grants from Boston Scientific. Dr Suradi reports consulting fees from Edwards Lifesciences, Medtronic, and Gore. Dr Sorajja reports institutional grant/contracts from Abbott Vascular and consulting fees from Boston Scientific Corporation, Edwards Lifesciences, and Xenter. Dr Szerlip serves as a Steering Committee member for Abbott Vascular; speaker and Advisory Board member for Boston Scientific Corporation; speaker for Edwards Lifesciences; and Steering Committee and Advisory Board member for Medtronic. Dr Dahle reports speaker and proctor fees from Boston Scientific Corporation and Medtronic and serves as principal trial investigator, speaker, and proctor for Edwards Lifesciences. Dr Makkar reports grant/contracts from Abbott Vascular and Edwards Lifesciences and consulting fees from Cordis Corporation and Medtronic. Dr Davidson reports institutional grant/contracts from Abbott Vascular and institutional grant/contracts and advisory roles with Edwards Lifesciences. Dr Sheth reports grant/contracts from Abbott Vascular and consulting fees and institutional grant/contracts from Edwards Lifesciences. Dr Shah reports consulting fees from 4C Medical Technologies, Abbott Structural, Adona Medical, Boston Scientific Corporation, CroiValve, Cultiv8, Edwards Lifesciences, Egg Medical, EvolutionMed, Foldax, GE Medical, Haemonetics, InQ8, Laza, Medtronic, Philips, Polares, W.L. Gore & Associates, vDyne, Unorthodox Ventures, Valcare Medical, and xDot. Dr Southard reports serving as a proctor and consultant for Edwards Lifesciences and consultant for Abbott Vascular and Shockwave Medical. Dr Depta reports consulting fees from Boston Scientific Corporation and Edwards Lifesciences. Dr Pop reports consulting fees from Edwards Lifesciences, Inari, and MurmurMD. Dr Guerrero reports institutional research grants from Edwards Lifesciences. Dr Pibarot reports institutional grant/contracts from Cardiac Success, Edwards Lifesciences, Medtronic, Novartis Pharma, and Pi-Cardiac. Dr Wang is an employee of Edwards Lifesciences. Dr Leon reports institutional grant/contracts from Abbott Fund, ABIOMED, Anora Heart, Inc, Boston Scientific Corporation, and Medtronic; consulting fees from Anteris, CroiValve, Foldax, Laminar, and MiRus LLC; serves as principal investigator and institutional grant/contract recipient for Edwards Lifesciences; and reports stock options from Pi-Cardia and Xenter MD and stock ownership in SoloPace. Dr Généreux reports consulting fees from 4C Medical, Abbott Vascular, ABIOMED, Haemonetics Corporation, Medtronic, Opsens, Inc, ShockWave Medical, and Teleflex, Inc; serves as principal investigator and consultant for Edwards Lifesciences; and reports consulting fees and stock options from Pi-Cardia, Puzzle Medical, Inc, and Saranas, Inc. The other authors report no conflicts.

### Supplemental Material

Supplemental Methods

Tables S1–S3

Figures S1–S5

## Supplementary Material
