# Supplementary material for: Age and Procedural Timing for Asymptomatic Severe Aortic Stenosis: Analysis From the EARLY TAVR Trial
Source: Circ Cardiovasc Interv. 2026 Jun 3;19(8):e016370. doi: 10.1161/CIRCINTERVENTIONS.125.016370 (PMC13652125; doi:10.1161/CIRCINTERVENTIONS.125.016370)
Supplement: Supplementary file 1 [file hcv-19-e016370-s001.pdf]

SUPPLEMENTAL MATERIAL

Age and Procedural Timing for Asymptomatic Severe Aortic Stenosis: Analysis from the EARLY TAVR trial

Kashish Goel et al.

Table of Contents

Supplemental Methods: 3

Figure S1: EARLY TAVR Patient Flow and Data Availability for Age Stratification Analysis 4

Figure S2: KM Curves for Clinical Outcomes Stratified by Age 5

Figure S3: Interaction Terms by Treatment and Age Quartiles for Composite Outcomes 6

Figure S4: KM Curves for Death, Stroke, or Unplanned CV Hospitalization Stratified by Age and Treatment 7

Figure S5: KM Curves for Death, Stroke, or HF Hospitalization Stratified by Age and Treatment 8

Table S1: Baseline Characteristics by Age and Treatment 9

Table S2: Procedural Characteristics Stratified by Age and Treatment 13

Table S3: Periprocedural Safety Outcomes Stratified by Age and Treatment 15

Supplemental Methods:

The first prespecified secondary endpoint of the EARLY TAVR trial was the composite of alive and well over 2 years was defined as being alive at 2 years with a Kansas City Cardiomyopathy Questionnaire (KCCQ) score of at least 75 that had not decreased more than 10 points from baseline. If a patient in the CS group had an AVR within 6 months of randomization, the pre-procedure KCCQ score was used; similarly, if a patient in the TAVR group had an AVR within 6 months of the procedure, the 30-day KCCQ score was used.

The second prespecified secondary endpoint of the EARLY TAVR trial was a composite of integrated measure of left ventricular (LV) health at 2 years, defined as an absolute LV global longitudinal strain ≥15%, a LV mass index <115 g/m^2 for men or < 95 g/m^2 for women, and a left atrial (LA) volume index ≤34 ml/m^2.[1]

Types of clinical presentation at the time of conversion in the CS arm included: advanced or acute signs and symptoms (acute valve syndrome), progressive signs and symptoms (progressive valve syndrome), and no symptoms (silent valve syndrome).[1]

Figure S1: EARLY TAVR Patient Flow and Data Availability for Age Stratification Analysis

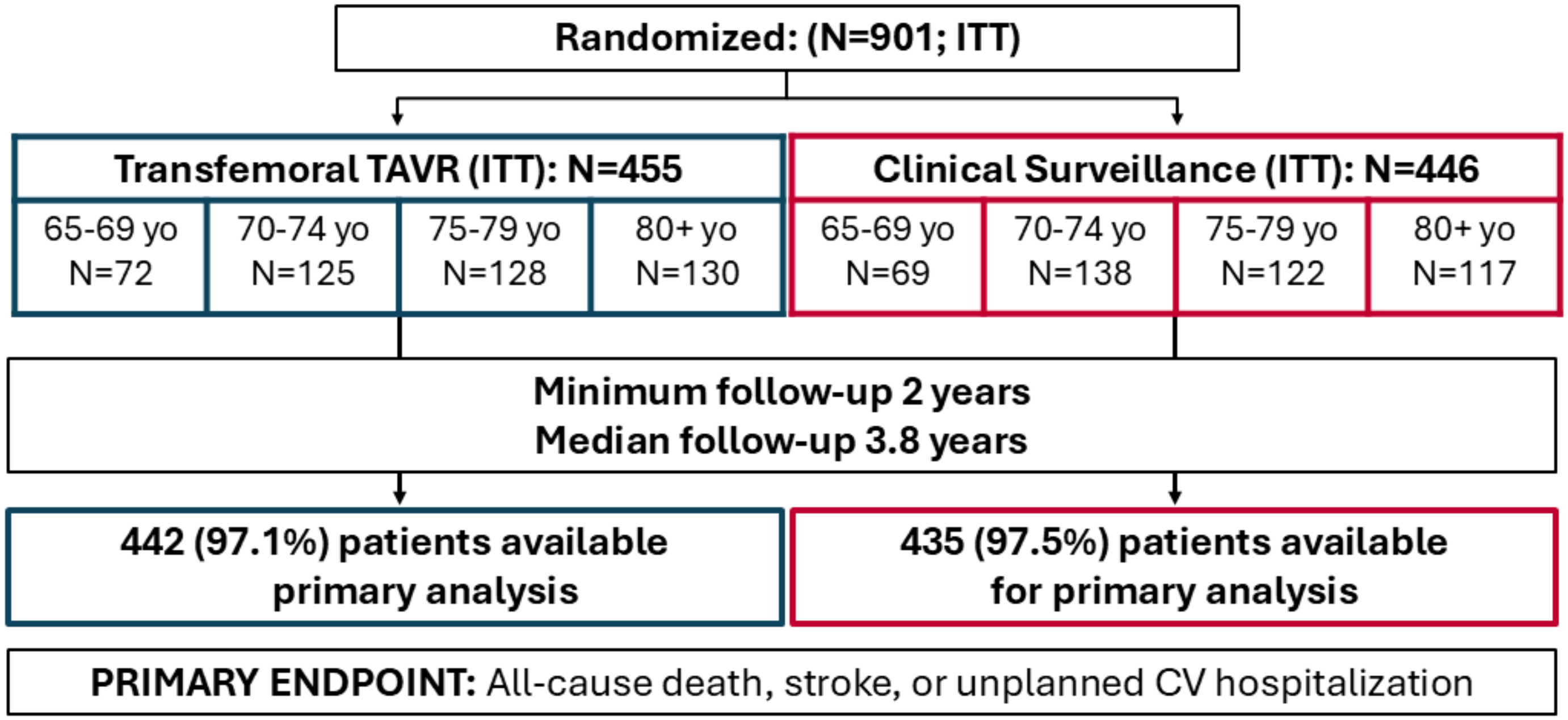

Patients in both treatment arms, TAVR and CS, were stratified into four age groups: 65-69, 70-74, 75-79, 80+.

Abbreviations: CS, clinical surveillance; TAVR, transcatheter aortic valve replacement.

Figure S2: KM Curves for Clinical Outcomes Stratified by Age

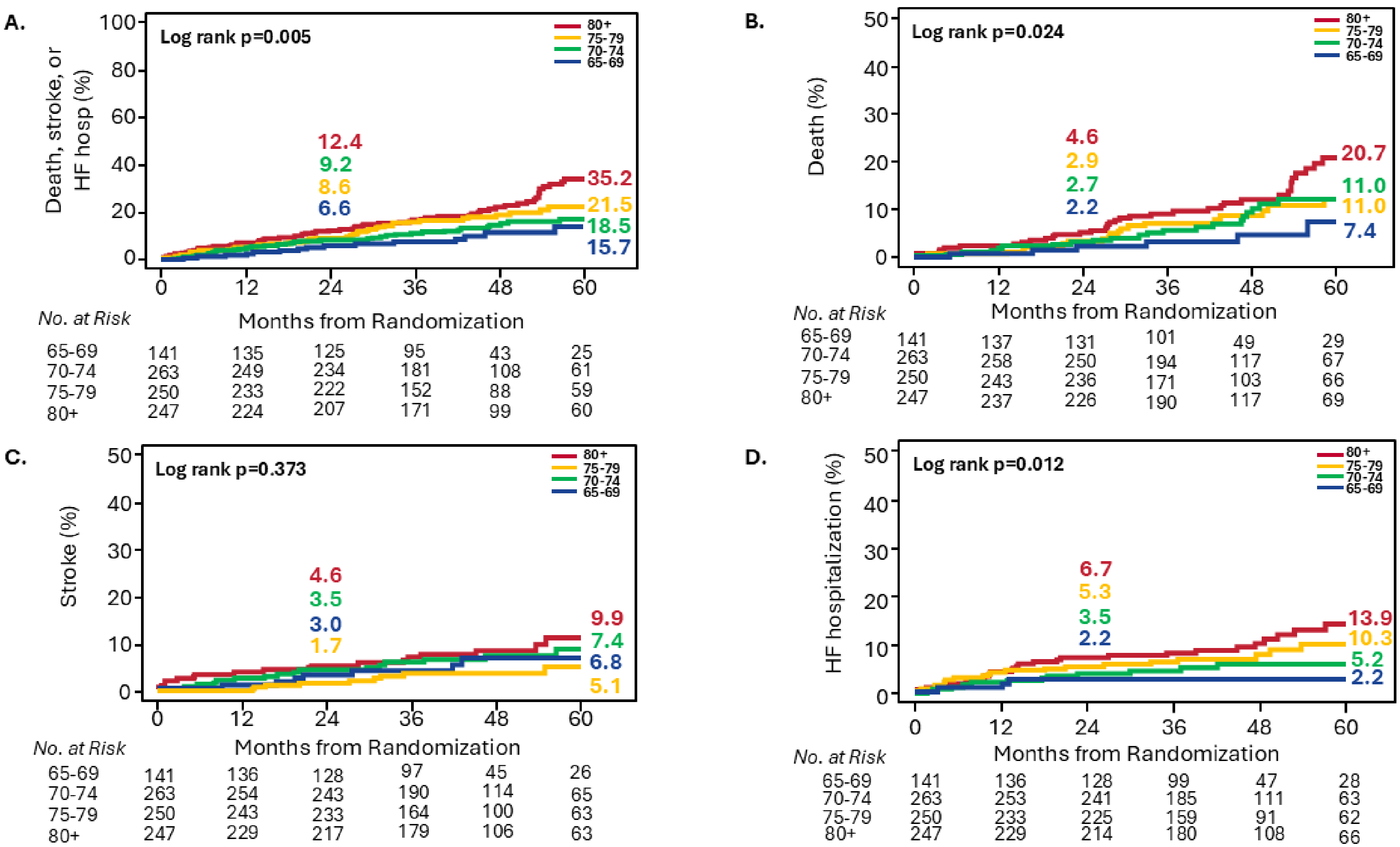

Kaplan-Meier curves for death (A), stroke (B), HF hospitalization (C), and the composite of death, stroke, or unplanned CV hospitalization (D) stratified by four age groups. P-value shown is based on an unadjusted Log-rank test with data up to 5 years. Age 65-69 is shown in blue, age 70-74 is shown in green, age 75-79 is shown in yellow, and age 80+ is shown in red. Abbreviations: CV, cardiovascular; HF, heart failure; KM, Kaplan-Meier.

Figure S3: Interaction Terms by Treatment and Age Quartiles for Composite Outcomes

ITT Population (N=901)

	Rate per 100 patient years TAVR	Rate per 100 patient years CS	Number Needed to Treat	HR [95% CI]	Interaction *p*-value
**Death, stroke, or unplanned CV hospitalization**					
Quartile 1	6.1	16.3	7	0.46 [0.25, 0.85]	
Quartile 2	16.4	32.3	5	0.43 [0.27, 0.67]	0.46
Quartile 3	15.1	36.0	4	0.40 [0.26, 0.63]	
Quartile 4	21.2	46.0	6	0.59 [0.40, 0.86]	
**Death, stroke, unplanned CV hosp, or intervention with advanced signs or symptoms**					
Quartile 1	5.8	22.3	6	0.28 [0.16, 0.51]	
Quartile 2	16.9	29.0	7	0.58 [0.37, 0.91]	0.10
Quartile 3	15.5	36.5	6	0.40 [0.26, 0.62]	
Quartile 4	21.2	46.4	6	0.63 [0.43, 0.92]	
**Death, stroke, or HF hospitalization**					
Quartile 1	1.7	5.2	24	0.35 [0.13, 0.95]	
Quartile 2	5.5	9.5	12	0.54 [0.29, 1.02]	0.65
Quartile 3	5.5	8.6	14	0.58 [0.30, 1.10]	
Quartile 4	7.5	13.5	23	0.70 [0.42, 1.19]	

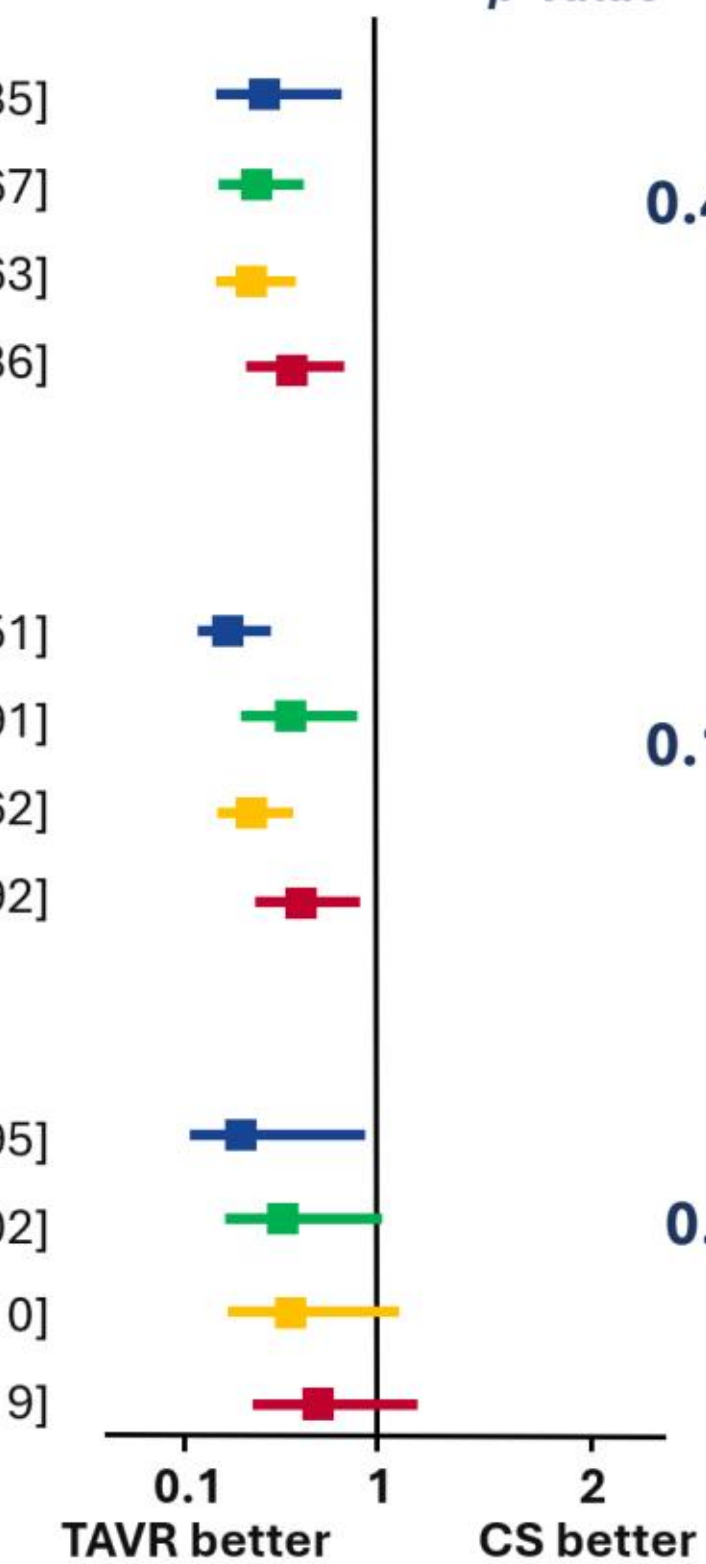

Forest plot showing clinical event rates per 100 patient years, number of patients needed to treat (NNT; calculated using 2-year Kaplan-Meier rate), risk and interaction p-values for 3 composite endpoints according to all intent-to-treat (ITT) population and four age groups. Clinical event hazard ratios and 95% confidence limits are based on Cox Proportional Hazard models with data up to 5 years. Interaction p-values reflect interaction between age group and treatment arm. Abbreviation: CV, cardiovascular.

Figure S4: KM Curves for Death, Stroke, or Unplanned CV Hospitalization Stratified by Age and Treatment

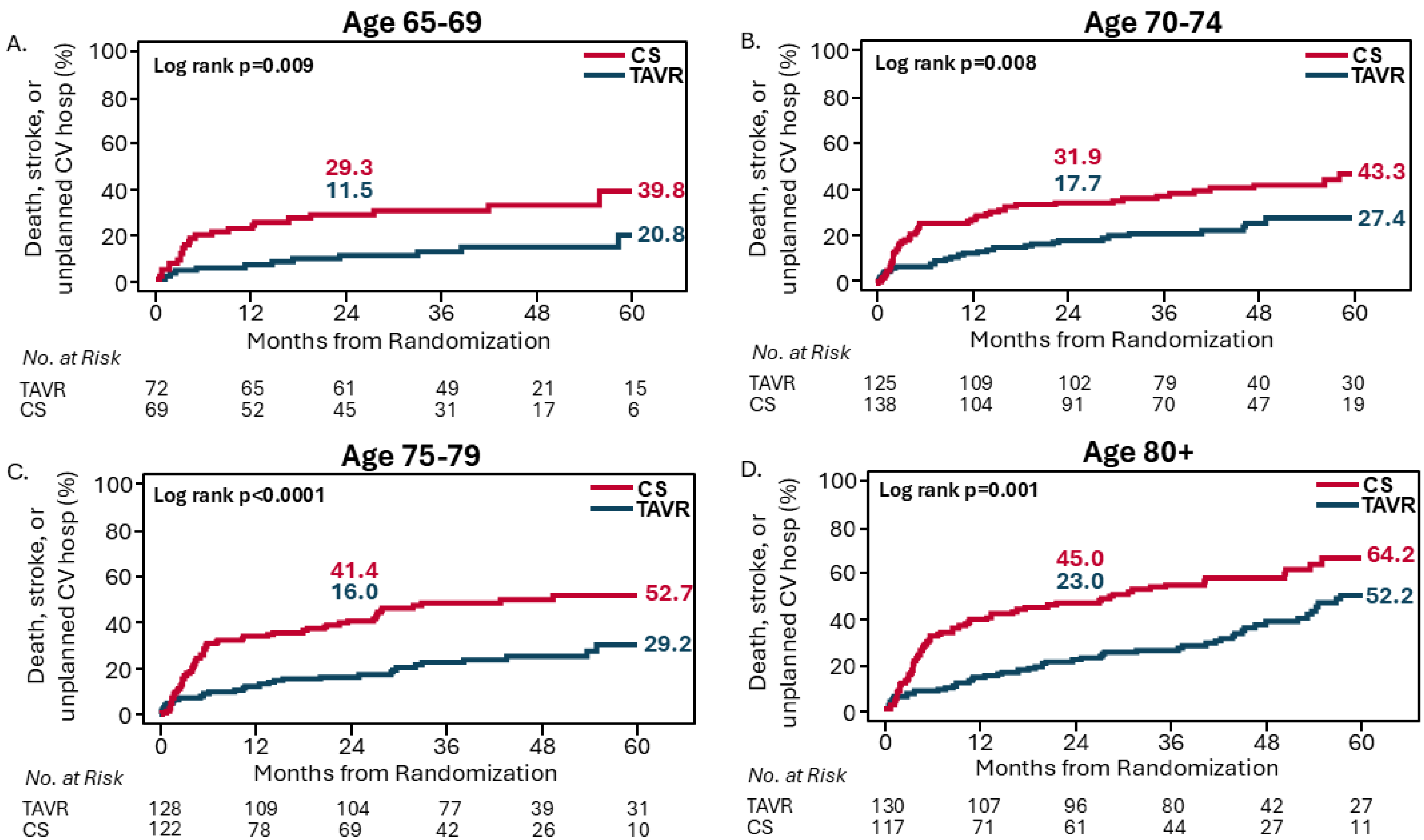

Kaplan-Meier curves for death, stroke, or unplanned CV hospitalization in age group 65-69 (A), age group 70-74 (B), age group 75-79 (C), and age group 80+ (D). P-value shown is based on an unadjusted Log-rank test with data up to 5 years. TAVR arm is shown in blue, CS arm is shown in red. Abbreviations: CS, clinical surveillance; CV, cardiovascular; TAVR, transcatheter aortic valve replacement.

Figure S5: KM Curves for Death, Stroke, or HF Hospitalization Stratified by Age and Treatment

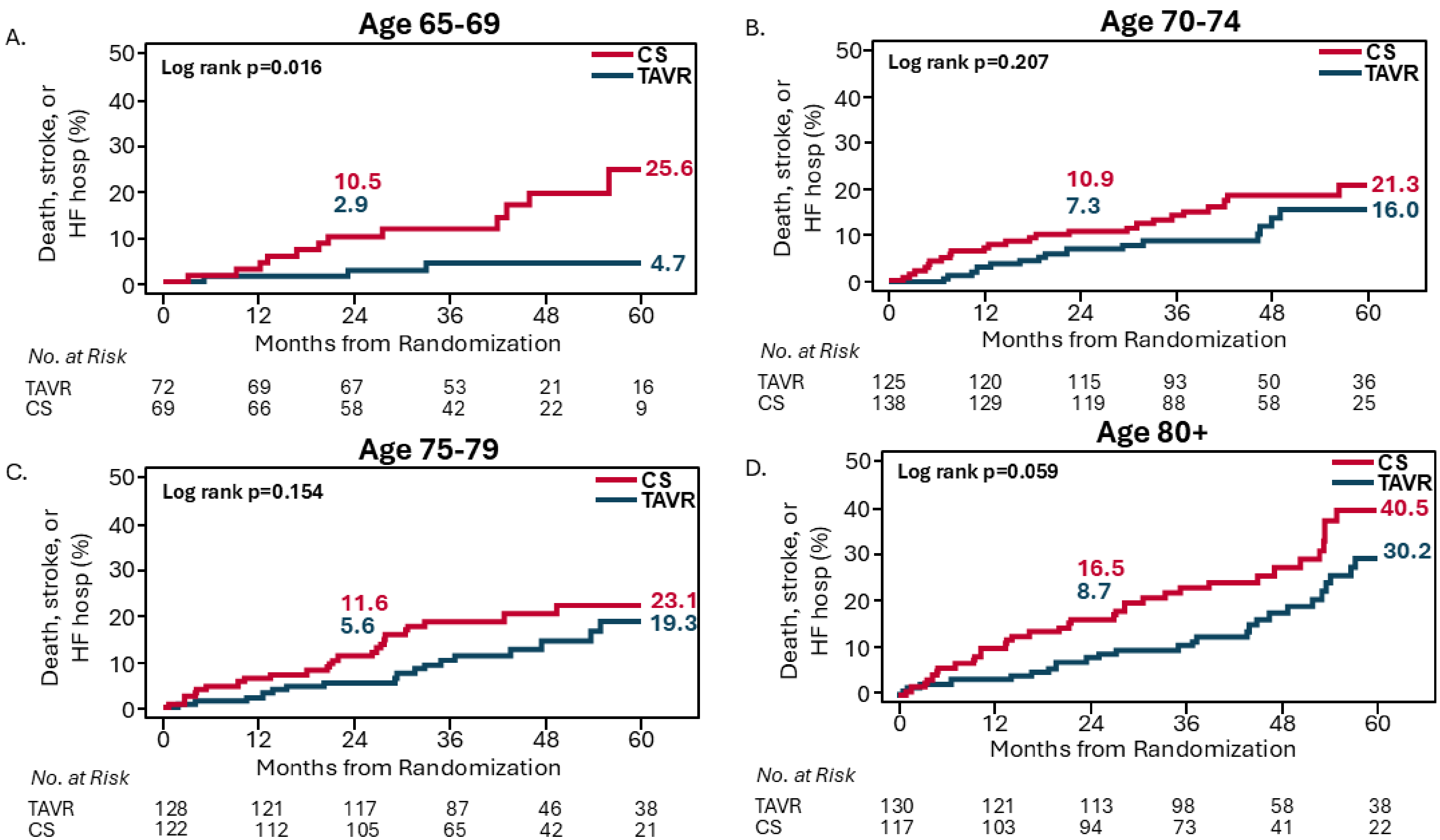

Kaplan-Meier curves for death, stroke, or HF hospitalization in age group 65-69 (A), age group 70-74 (B), age group 75-79 (C), and age group 80+ (D). P-value shown is based on an unadjusted Log-rank test with data up to 5 years. TAVR arm is shown in blue, CS arm is shown in red. Abbreviations: CS, clinical surveillance; HF, heart failure; TAVR, transcatheter aortic valve replacement.

Table S1: Baseline Characteristics by Age and Treatment

Characteristic	Age 65-69 (N=141)			Age 70-74 (N=263)			Age 75-79 (N=250)			Age 80+ (N=247)		
	TAVR (N=72)	CS (N=69)	P-value	TAVR (N=125)	CS (N=138)	P-value	TAVR (N=128)	CS (N=122)	P-value	TAVR (N=130)	CS (N=117)	P-value
Age (years)	67.2 ± 1.35	67.1 ± 1.55	---	72.4 ± 1.31	71.9 ± 1.38	---	77.0 ± 1.43	76.9 ± 1.39	---	83.4 ± 3.23	83.4 ± 2.90	---
Male	70.8% (51/72)	69.6% (48/69)	>0.99	66.4% (83/125)	69.6% (96/138)	0.60	73.4% (94/128)	64.8% (79/122)	0.17	73.8% (96/130)	65.0% (76/117)	0.17
Race -White	93.1% (67/72)	89.9% (62/69)	0.16	95.2% (119/125)	96.4% (133/138)	0.79	95.3% (122/128)	93.4% (114/122)	0.77	98.5% (128/130)	96.6% (113/117)	0.71
Body mass index (kg/m^2)	31.2 ± 5.71	29.8 ± 5.13	0.10	28.3 ± 4.63	29.6 ± 5.09	0.05	28.3 ± 3.71	28.2 ± 4.36	0.74	27.1 ± 4.15	27.1 ± 4.07	0.66
Comorbidities												
STS risk score	1.2 ± 0.54	1.2 ± 0.66	0.26	1.6 ± 0.81	1.4 ± 0.76	0.16	1.8 ± 0.73	1.7 ± 0.75	0.16	2.5 ± 1.22	2.4 ± 1.10	0.96
Able to perform treadmill stress test	87.5% (63/72)	92.8% (64/69)	0.40	95.2% (119/125)	92.8% (128/138)	0.45	93.0% (119/128)	89.3% (109/122)	0.45	84.6% (110/130)	88.9% (104/117)	0.35
KCCQ Overall Score	92.2 ± 7.59	91.1 ± 11.03	0.88	93.8 ± 9.11	92.5 ± 9.94	0.12	92.9 ± 8.31	93.7 ± 8.80	0.12	91.9 ± 9.23	92.6 ± 8.29	0.62

Characteristic	Age 65-69 (N=141)			Age 70-74 (N=263)			Age 75-79 (N=250)			Age 80+ (N=247)		
	TAVR (N=72)	CS (N=69)	P-value	TAVR (N=125)	CS (N=138)	P-value	TAVR (N=128)	CS (N=122)	P-value	TAVR (N=130)	CS (N=117)	P-value
Atrial fibrillation	2.8% (2/72)	5.8% (4/69)	0.44	13.6% (17/125)	8.7% (12/138)	0.24	19.5% (25/128)	13.9% (17/122)	0.24	20.8% (27/130)	22.2% (26/117)	0.88
Coronary artery disease	20.8% (15/72)	18.8% (13/69)	0.83	24.0% (30/125)	26.8% (37/138)	0.67	35.9% (46/128)	23.0% (28/122)	0.67	32.3% (42/130)	29.9% (35/117)	0.78
Myocardial infarction	1.4% (1/72)	5.8% (4/69)	0.20	4.0% (5/125)	2.9% (4/138)	0.74	3.9% (5/128)	4.9% (6/122)	0.74	9.2% (12/130)	3.4% (4/117)	0.07
PVD	6.9% (5/72)	2.9% (2/69)	0.44	9.6% (12/125)	3.6% (5/138)	0.08	7.8% (10/128)	6.6% (8/122)	0.08	4.6% (6/130)	5.1% (6/117)	>0.99
Prior stroke	1.4% (1/72)	1.4% (1/69)	>0.99	5.6% (7/125)	5.8% (8/138)	>0.99	3.1% (4/128)	5.7% (7/122)	>0.99	5.4% (7/130)	3.4% (4/117)	0.55
COPD	2.8% (2/72)	0.0% (0/69)	0.50	2.4% (3/125)	5.8% (8/138)	0.22	3.9% (5/128)	4.1% (5/122)	0.22	2.3% (3/130)	1.7% (2/117)	>0.99
Diabetes	33.3% (24/72)	26.1% (18/69)	0.36	28.8% (36/125)	29.7% (41/138)	0.89	23.4% (30/128)	30.3% (37/122)	0.89	22.3% (29/130)	15.4% (18/117)	0.20
NT-pro-BNP, pg/mL	277.8 ± 354.32 (60)	277.8 ± 376.11 (62)	0.94	431.9 ± 621.11 (114)	385.9 ± 470.07 (112)	0.90	588.8 ± 722.22 (120)	602.5 ± 735.48 (105)	0.90	697.4 ± 773.70 (120)	786.2 ± 1076.11 (105)	0.98
Creatinine (mg/dL)	0.9 ± 0.19 (72)	0.9 ± 0.19 (69)	0.23	0.9 ± 0.25 (125)	1.0 ± 0.25 (138)	0.28	1.0 ± 0.27 (128)	1.0 ± 0.24 (122)	0.28	1.1 ± 0.31 (130)	1.0 ± 0.26 (116)	0.01

Characteristic	Age 65-69 (N=141)			Age 70-74 (N=263)			Age 75-79 (N=250)			Age 80+ (N=247)		
	TAVR (N=72)	CS (N=69)	P-value	TAVR (N=125)	CS (N=138)	P-value	TAVR (N=128)	CS (N=122)	P-value	TAVR (N=130)	CS (N=117)	P-value
Hypertension	72.2% (52/72)	75.4% (52/69)	0.71	82.4% (103/125)	80.4% (111/138)	0.75	79.7% (102/128)	84.4% (103/122)	0.75	86.2% (112/130)	84.6% (99/117)	0.86
Echo characteristics												
AoV peak velocity (m/s)	4.3 ± 0.49 (72)	4.4 ± 0.45 (68)	0.91	4.3 ± 0.39 (124)	4.4 ± 0.44 (136)	0.24	4.4 ± 0.50 (128)	4.4 ± 0.41 (121)	0.39	4.3 ± 0.43 (127)	4.3 ± 0.45 (116)	0.26
Mean gradient (mmHg)	47.5 ± 10.66 (72)	47.7 ± 11.19 (68)	0.84	46.6 ± 9.20 (124)	47.5 ± 10.15 (136)	0.14	47.4 ± 11.32 (128)	47.6 ± 10.16 (121)	0.54	45.1 ± 9.15 (127)	46.6 ± 11.32 (117)	0.26
AoV area (cm^2)	0.89 ± 0.178 (70)	0.89 ± 0.148 (66)	0.76	0.87 ± 0.318 (120)	0.87 ± 0.345 (131)	0.89	0.85 ± 0.192 (123)	0.82 ± 0.153 (114)	0.30	0.83 ± 0.162 (123)	0.82 ± 0.162 (114)	0.83
LVEF (%)	68.0 ± 6.58 (72)	66.6 ± 6.36 (69)	0.18	67.4 ± 6.24 (124)	67.5 ± 5.95 (137)	0.85	67.2 ± 6.68 (128)	67.6 ± 7.17 (121)	0.51	67.3 ± 6.74 (127)	67.4 ± 7.17 (117)	0.64
LV mass index (g/m^2)	102.7 ± 20.05 (72)	107.0 ± 26.69 (69)	0.65	98.6 ± 20.65 (123)	100.2 ± 25.60 (136)	0.88	103.3 ± 24.87 (123)	103.6 ± 20.53 (118)	0.44	104.4 ± 24.75 (124)	102.2 ± 25.06 (117)	0.48

LA volume index (mL/m^2)	32.8 ± 8.77 (71)	35.1 ± 9.78 (68)	0.14	35.5 ± 9.00 (124)	36.4 ± 11.31 (136)	0.80	38.4 ± 9.89 (128)	37.4 ± 9.53 (120)	0.49	39.0 ± 11.54 (127)	40.5 ± 16.03 (115)	0.41
Bicuspid	20.8% (15/72)	26.1% (18/69)	0.55	8.8% (11/125)	8.7% (12/138)	>0.99	6.3% (8/128)	5.7% (7/122)	>0.99	2.3% (3/130)	1.7% (2/115)	>0.99

Categorical measures presented as % (n/Total n); continuous measures presented as mean ± STD (n). P value is from Fisher's Exact test for categorical variables, and from Wilcox Sum test for continuous variables.

Abbreviations: AoV, aortic valve; COPD, chronic obstructive pulmonary disease; CS, clinical surveillance; KCCQ, Kansas City Cardiomyopathy Questionnaire; LA, left atrial; LV, left ventricular; LVEF, NT-pro-BNP, N-terminal pro b-type natriuretic peptide; PVD, peripheral vascular disease; STS, Society of Thoracic Surgeons; TAVR, transcatheter aortic valve replacement.

Table S2: Procedural Characteristics Stratified by Age and Treatment

Parameter	Age 65-69 (N=132)		Age 70-74 (N=247)		Age 75-79 (N=226)		Age 80+ (N=227)	
	TAVR (N=70)	CS with DAVR (N=62)	TAVR (N=124)	CS with DAVR (N=123)	TAVR (N=124)	CS with DAVR (N=102)	TAVR (N=126)	CS with DAVR (N=101)
Anesthesia duration (min)	116.4 ± 5.13 (70)	125.1 ± 6.41 (62)	118.0 ± 5.33 (124)	113.2 ± 3.51 (122)	116.3 ± 3.81 (124)	123.9 ± 4.23 (101)	117.8 ± 3.19 (126)	118.2 ± 5.69 (101)
Procedure duration (min)	38.8 ± 2.02 (70)	48.7 ± 4.29 (62)	41.2 ± 1.60 (124)	41.2 ± 1.86 (120)	41.1 ± 1.72 (123)	47.1 ± 5.84 (99)	38.3 ± 1.47 (126)	45.2 ± 3.72 (99)
Total fluoroscopy time (min)	12.1 ± 0.79 (70)	15.7 ± 1.08 (59)	13.0 ± 0.61 (122)	13.1 ± 0.62 (119)	13.4 ± 0.62 (124)	13.1 ± 0.49 (98)	12.6 ± 0.51 (126)	14.2 ± 0.76 (98)
BAV performed	31.4% (22/70)	42.4% (25/59)	32.3% (40/124)	30.0% (36/120)	25.0% (31/124)	31.4% (32/102)	25.4% (32/126)	35.0% (35/100)
Post dilatation performed	8.6% (6/70)	11.9% (7/59)	19.4% (24/124)	15.8% (19/120)	12.1% (15/124)	11.9% (12/101)	13.5% (17/126)	14.0% (14/100)
Cerebral protection device used	17.1% (12/70)	28.8% (17/59)	21.8% (27/124)	31.7% (38/120)	25.8% (32/124)	24.5% (25/102)	19.8% (25/126)	22.0% (22/100)
Index hospitalization stay (days)*	1.1 ± 0.06 (70)	1.6 ± 0.25 (62)	1.3 ± 0.08 (124)	1.5 ± 0.22 (123)	1.5 ± 0.20 (124)	1.3 ± 0.07 (102)	1.5 ± 0.09 (126)	2.0 ± 0.47 (101)
ICU stay (days)†	0.25 ± 0.062 (70)	0.35 ± 0.149 (62)	0.33 ± 0.065 (124)	0.48 ± 0.104 (123)	0.55 ± 0.164 (124)	0.30 ± 0.058 (102)	0.44 ± 0.060 (126)	0.96 ± 0.487 (101)

Categorical measures presented as % (n/Total n); continuous measures presented as mean ± STD (n).
*Index hospitalization days = discharge date – procedure date.
†ICU stays (days) = sum of all ICU stay duration in hours/24; subjects with no ICU stay have duration of 0 day.

Abbreviations: BAV, balloon aortic valvuloplasty; CS, clinical surveillance; DAVR, delayed aortic valve replacement; ICU, intensive care unit; SE, standard error; TAVR, transcatheter aortic valve replacement.

Table S3: Periprocedural Safety Outcomes Stratified by Age and Treatment

Parameter	Age 65-69 (N=132)		Age 70-74 (N=247)		Age 75-79 (N=226)		Age 80+ (N=227)	
	TAVR (N=70)	CS with DAVR (N=62)	TAVR (N=124)	CS with DAVR (N=123)	TAVR (N=124)	CS with DAVR (N=102)	TAVR (N=126)	CS with DAVR (N=101)
All cause death	0 (0.0)	0 (0.0)	0 (0.0)	0 (0.0)	1 (0.8)	0 (0.0)	0 (0.0)	0 (0.0)
Cardiovascular death	0 (0.0)	0 (0.0)	0 (0.0)	0 (0.0)	0 (0.0)	0 (0.0)	0 (0.0)	0 (0.0)
Non-cardiovascular death	0 (0.0)	0 (0.0)	0 (0.0)	0 (0.0)	1 (0.8)	0 (0.0)	0 (0.0)	0 (0.0)
Stroke	0 (0.0)	2 (1.6)	0 (0.0)	2 (1.6)	0 (0.0)	0 (0.0)	4 (3.2)	4 (4.0)
Disabling stroke	0 (0.0)	0 (0.0)	0 (0.0)	2 (1.6)	0 (0.0)	0 (0.0)	0 (0.0)	2 (2.0)
Non-disabling stroke	0 (0.0)	2 (1.6)	0 (0.0)	0 (0.0)	0 (0.0)	0 (0.0)	4 (3.2)	2 (2.0)
New onset atrial fibrillation*	1 (1.5)	0 (0.0)	3 (2.8)	3 (2.9)	9 (9.1)	4 (4.5)	4 (4.0)	3 (4.1)
Major vascular complications	1 (1.4)	2 (1.6)	1 (0.8)	1 (0.8)	2 (1.6)	1 (1.0)	2 (1.6)	1 (1.0)
Bleeding complications	9 (12.9)	11 (17.7)	11 (7.3)	15 (12.2)	21 (14.5)	17 (14.7)	22 (16.7)	15 (11.9)

Table S3: Periprocedural Safety Outcomes Stratified by Age and Treatment

Parameter	Age 65-69 (N=132)		Age 70-74 (N=247)		Age 75-79 (N=226)		Age 80+ (N=227)	
	TAVR (N=70)	CS with DAVR (N=62)	TAVR (N=124)	CS with DAVR (N=123)	TAVR (N=124)	CS with DAVR (N=102)	TAVR (N=126)	CS with DAVR (N=101)
Myocardial infarction	1 (1.4)	0 (0.0)	1 (0.8)	0 (0.0)	0 (0.0)	1 (1.0)	0 (0.0)	1 (1.0)
Acute kidney injury†	0 (0.0)	2 (3.2)	2 (1.6)	4 (3.3)	6 (4.8)	2 (2.0)	3 (2.4)	5 (5.0)
Coronary obstruction requiring intervention	0 (0.0)	0 (0.0)	0 (0.0)	0 (0.0)	0 (0.0)	0 (0.0)	0 (0.0)	0 (0.0)
New permanent pacemaker‡	1 (1.4)	4 (6.6)	5 (4.1)	10 (7.5)	6 (5.2)	8 (7.9)	12 (10.3)	11 (11.2)

Data presented as numbers of events (Kaplan-Meier estimates); Periprocedural defined as ≤30 days from index procedure in the TAVR arm or date of conversion to AVR in the CS arm.

*New onset fibrillation is only estimated among those without atrial fibrillation at baseline (for CS group VI population, "baseline" refers to pre-procedure visit).

†Acute kidney injury is from site reported data.

‡New pacemaker is only estimated among those without pacemaker at baseline (for CS group VI population, "baseline" refers to pre-procedure visit).

Abbreviations: AVR, aortic valve replacement; CS, clinical surveillance; TAVR, transcatheter aortic valve replacement; VI, valve implant.
